# Acute Toxicity Investigation and Anti-diarrhoeal Effect of the Chloroform-Methanol Extract of the Leaves of *Persea americana*

**Published:** 2014

**Authors:** Odo Christian E, Nwodo Okwesili FC, Joshua Parker E, Ugwu Okechukwu PC

**Affiliations:** *Pharmacology Research Unit, Department of Biochemistry, University of Nigeria, Nsukka, Nigeria.*

**Keywords:** Luraceae, Acute toxicity, Enteropooling, Hyoscine butylbromide, Intestinal contents

## Abstract

*Persea americana* is a plant used by traditional medicine practitioners to treat ailments including diarrhoea and diabetes mellitus in Nigeria. Hence, the chloroform and the methanol fractions of the chloroform-methanol extract of the leaves of *P. americana* were evaluated for their acute toxicity as well as anti-diarrhoeal effects in Wistar rats to substantiate this claim. The chloroform and methanol fractions [at graded doses of 100 and 200 mg/Kg body weight (b.w) of each] were studied for their anti-diarrhoeal effects in terms of the reductions in the wetness of faeces and the frequency of defaecation of castor oil-induced diarrhoea. To understand the mechanism of their anti-diarrhoeal effects, their actions were further evaluated on castor oil-induced enteropooling (intestinal fluid accumulation). The median lethal dose (LD_50_) of the methanol fraction was found to be less than 5000 mg/Kg b.w. At the two doses, the chloroform and the methanol fractions showed dose-dependent significant (p < 0.05) reductions in the wetness of faeces and the frequency of defaecation with the 200 mg/Kg b.w of the chloroform fraction being the most effective. Results of the fractions were comparable with those of the standard anti-diarrhoeal drug, hyoscine butylbromide (3 mg/Kg b.w). Both fractions produced remarkable (p < 0.05) dose-related inhibition of castor oil-induced enteropooling as shown by the significant (p < 0.05) decreases in the weight and volume of the intestinal contents. Experimental findings show that the chloroform-methanol extract of the leaves of *P. americana* possesses significant anti-diarrhoeal effect and may be a potent source of anti-diarrhoeal drug(s) in future.

## Introduction

Millions of people in developing countries, for instance Nigeria, use herbal medicines because they are locally available and are prescribed by traditional medicine practitioners who are a part of their community. About 80 percent of the world population relies on the use of traditional medicine, which is predominantly based on plant material (WHO, 1993). Over 90 percent of Nigerians in the rural areas and 40 percent in the urban areas depend partly or wholly on traditional medicine for their health care (Alabi *et al*., 2005). The use of herbal medicines as complements or alternatives to orthodox medicines has been on the increase. The reasons, which have given rise to this trend, include: cheapness, availability and accessibility of these natural medicines (Larrey, 1994). On the other hand, their use is limited because many of the claimed medicinal values have not been scientifically evaluated and their safety profiles uncertain (Ernst, 2005).

Diarrhoea is an alteration in the normal bowel movement, characterised by increase in the water content in the intestine and/or frequency of stools (Alam and Ashra, 2003). Diarrhoea has also been defined by the World Health Organisation (2004), as having three or more loose or liquid stools per day, or as having more stools than is normal for a person. Diarrhoea can lead to severe dehydration and become life-threatening when not treated (Alam and Ashra, 2003). In developing countries, diarrhoea, which may or may not be infectious, is one of the leading causes of morbidity and mortality in children and one out of every five children dies of diarrhoea before the age of five. Each year, more than five million people, die of diarrhoea, 80% of who are children less than one year of age (Nester *et al*., 1998). The major causative agents of diarrhoea in man include: *Shigella flexneri*, *Staphylococcus aureus*, *Escherichia coli*, *Salmonella typhi *and *Candida albicans *(Anne and Geboes, 2002). Antibiotics are the major remedy for infectious diseases including diarrhoea; however, significant increase in the resistance to antibiotics has been observed in common human pathogens worldwide (Hellinger, 2000). Similarly, oral rehydration therapy (ORT) is a key factor in the decline of child mortality due to diarrhoea (Sastry and Burgard, 2005). In addition, the incidence of the disease has remained unchanged and this treatment (ORT) often fails in a state of high stool output (Brijesh *et al*., 2006). In view of this, there is the need to search for plants with anti-diarrhoeal effect.

*Persea americana *(avocado or alligator pear) is an almost evergreen tree belonging to the laurel family, Lauraceae. It is indigenous to Central and South America but is now cultivated in the United States, Asia, parts of Europe and tropical Africa. The plant is a tall evergreen tree that can grow up to 65 feet in height. The leaves are alternate, dark green and glossy on the upper surface, whitish on the underside; variable in shape (lanceolate, elliptic, oval, ovate or obovate) and 7.5 – 40 cm long. The aqueous leaf extract, for example, has analgesic and anti-inflammatory (Adeyemi *et al*., 2002), anti-convulsant (Ojewole and Amabeoku, 2006), hypoglycaemic and hypocholesterolaemic (Brai *et al*., 2007), vasorelaxant and blood pressure-reducing, activities in animal studies. It is alleged to stimulate and regulate menstruation. The leaf decoction is taken as a remedy for diarrhoea, sore throat and haemorrhage (Brai *et al*., 2007). The present study was undertaken to evaluate the acute toxicity and anti-diarrhoeal effect of the chloroform-methanol extract of the leaves of *P. americana* in castor oil-induced diarrhoeal rats.

## Experimental

*The plant*

Fresh leaves of *P. americana* were got from their trees at various points in Iheakpu-Awka, Igbo Eze South Local Government Area of Enugu State, Nigeria. The leaves were identified by Mr. A. Ozioko of Bioresource Development and Conservation Programme (BDCP) Research Centre, Nsukka. 

*Preparation of the extract*

Fresh leaves of *P. americana* were plucked and washed with distilled water. The leaves were spread on a clean mat in a well-ventilated room with regular turning to enhance even drying and avoid decaying. The leaves were shade-dried for 3 weeks. The shade-dried leaves were pulverised with an electric blender and a known weight (1380 g) of the pulverised *P. americana* leaves was macerated in 5 volumes (w/v) of chloroform-methanol (2:1) for 24 hours. The mixture was separated with Whatman No 1 filter paper. The filtrate of the macerate was shaken with distilled water that measured 20 percent its volume to obtain two (2) fractions. The upper fraction (methanol fraction) was separated from the lower fraction (chloroform fraction). The methanol and the chloroform fractions were concentrated in a rotary evaporator, dried in a boiling water bath and weighed. 

*Animals*

A total of 70 adult albino Wistar rats of between 8 and 12 weeks old with average weight of 125 ± 25 g and 48 albino mice weighing 25 ± 5 g were obtained from the Animal house of the Faculty of Pharmaceutical Sciences, University of Nigeria, Nsukka. The rats were acclimatised for one week under a standard environmental condition with a 12 h light and dark cycle and maintained on a regular feed and water *ad libitum*. The Principles of Laboratory Animal Care were adhered to. The experimental protocol was approved by the University Animal Research Ethical Committee.

*Acute toxicity study*

The acute toxicity and lethality (LD_50_) of the methanol and the chloroform fractions were determined using mice according to slightly modified method of Lorke (1983).

*Chemicals and reagents*

The chemicals and reagents used for this study were of analytical grade and procured from reputable scientific shops at Nsukka. They included the following: hyoscine butylbromide [standard anti-diarrhoeal drug (Sigma-Aldrich, Inc., St. Louis, USA)], methanol and chloroform (both supplied by BDH Chemicals *Ltd*., Poole, England), castor oil (laxative) and 3 percent (v/v) tween 80 (vehicle for dissolving the extract). 

*Castor oil-induced diarrhoea test*

Castor oil-induced diarrhoea was evaluated with 35 rats using the methods of Awouters *et al*. (1978) and Nwodo and Alumanah (1991) with slight modification.

*Castor oil-induced enteropooling test*

Castor oil-induced enteropooling was determined with 35 rats by the method of Robert *et al*. (1976).

*Statistical analysis*

The data obtained from the study were subjected to one-way analysis of variance (ANOVA). Significant differences were observed at p ≤ 0.05. The results were expressed as means of five replicates ± standard deviations (SD). This analysis was done using the computer software known as Statistical Package for Social Sciences (SPSS), version 16.

## Results

*The acute toxicity and lethality (LD*_50_ *) of the methanol and the chloroform fractions*

As shown in Tables 1 and 2, the result of this investigation shows that there was no lethality or any sign of toxicity in the four groups of three mice each that received 10, 100, 1000 mg/Kg body weight of each fraction of the chloroform-methanol extract of the leaves of *P. americana* and 5 mL/Kg body weight of 3% v/v tween 80 respectively at the end of the first phase of the study. At the end of the second phase of the study, there was neither death nor obvious sign of toxicity in the groups of mice that received 1900 and 2600 mg/Kg body weight of each fraction of the chloroform-methanol extract of the leaves of *P. americana*. However, there were death and obvious signs of toxicity (such as sluggishness, swollen face and eyes) in the groups of mice administered 5000 mg/Kg body weight of the methanol and the chloroform fractions respectively within 24 hours of administration (Tables 3 and 4).

**Table 1:** The first phase of the acute toxicity and lethality (LD_50_) of the methanol fraction

**Groups of the mice**	**Doses of the vehicle (tween 80) and the methanol fraction**	**Mortality**
Group 1(Solvent control)	5 mL/Kg of 3% v/v tween 80	0/3
Group 2	10 mg/Kg	0/3
Group 3	100 mg/Kg	0/3
Group 4	1000 mg/Kg	0/3

**Table 2:** The first phase of the acute toxicity and lethality (LD_50_) of the chloroform fraction

**Groups of the mice**	**Doses of the vehicle (tween 80) and the chloroform fraction **	**Mortality**
Group 1(Solvent control)	5 mL/Kg of 3% v/v tween 80	0/3
Group 2	10 mg/Kg	0/3
Group 3	100 mg/Kg	0/3
Group 4	1000 mg/Kg	0/3

**Table 3:** The second phase of the acute toxicity and lethality (LD_50_) of the methanol fraction.

**Groups of the mice**	**Doses of the vehicle (tween 80) and the methanol fraction **	**Mortality**
Group 1(Solvent control)	5 mL/Kg of 3% v/v tween 80	0/3
Group 2	1600 mg/Kg	0/3
Group 3	2900 mg/Kg	0/3
Group 4	5000 mg/Kg	2/3

**Table 4:** The second phase of the acute toxicity and lethality (LD_50_) of the chloroform fraction

**Groups of the mice**	**Doses of the vehicle (tween 80) and the chloroform fraction **	**Mortality**
Group 1(Solvent control)	5 mL/Kg of 3% v/v tween 80	0/3
Group 2	1600 mg/Kg	0/3
Group 3	2900 mg/Kg	0/3
Group 4	5000 mg/Kg	1/3

*Effects of the methanol and the chloroform fractions on castor oil-induced diarrhoea in terms of the wetness of faeces*

In the castor oil-induced diarrhoea experiment (wetness of faeces test), the rats in the group that received neither castor oil nor any of the fractions of the chloroform-methanol extract of the leaves of *P. americana* (group 1) had significantly (p < 0.05) decreased numbers of wet faeces (0.00 ± 0.00, 0.25 ± 0.50, 0.25 ± 0.50 and 0.00 ± 0.00) at the first, second, third and fourth hours of post-treatment respectively when compared to the values (1.50 ± 1.29, 2.00 ± 0.00, 2.00 ± 1.41 and 1.50 ± 0.58) obtained for rats in the castor oil-treated control group (group 2) as shown in Table 5. The chloroform fraction of the extract at the dose of 200 mg/Kg body weight, in a similar manner as the standard anti-diarrhoeal agent (hyoscine butylbromide), inhibited significantly (p < 0.05) the wetness of faeces of rats in group 7 as evidenced by the significant (p < 0.05) reduction in the number of wet faeces of rats in group 7 at the third and fourth hours of post treatment (0.50 ± 0.82 and 0.50 ± 0.58 respectively) when compared to the values (2.00 ± 1.41 and 1.50 ± 0.58) obtained for rats in the castor oil-treated control group (group 2). Both fractions of the extract at the tested doses decreased, in a dose-related manner, the wetness of faeces of rats in groups 4, 5, 6 and 7 at the first, second, third and fourth hours of post treatment when compared to those of the rats in group 2 as shown in Table 5.

**Table 5:** Effects of the chloroform and the methanol fractions on the wetness of faeces

**Groups**	**Treatments**	**Number of wet faeces after the**			
		**1** ^st^ ** hour**	**2** ^nd^ ** hour**	**3** ^rd^ ** hour**	**4** ^th^ ** hour**
1	5 mL/Kg of 3% v/v tween 80 (vehicle) only	0.00 ± 0.00^a^	0.25 ± 0.50^a^	0.25 ± 0.50^a^	0.00 ± 0.00^a^
2	Vehicle + 1 mL per oral (p.o) of castor oil (CO)	1.50 ±1.29^b^	2.00 ± 0.00^b^	2.00 ± 1.41^b^	1.50 ± 0.58^b^
3	3 mg/Kg of hyoscine + 1 mL p.o of CO	0.50 ± 0.58^b^	1.00 ± 0.00^c^	0.50 ± 0.58^c^	0.00 ± 0.00^a^
4	100 mg/kg of methanol fraction + 1 ml p.o of CO	1.50 ± 1.00^b^	1.75 ± 0.50^b^	0.75 ± 0.96^b^	1.25 ± 0.96^b^
5	200 mg/Kg of methanol fraction + 1 mL p.o of CO	1.50 ± 0.58^b^	1.75 ± 0.96^b^	1.00 ± 0.82^b^	0.75 ± 0.96^b^
6	100 mg/Kg of chloroform fraction +1 mL p.o of CO	1.50 ± 1.29^b^	1.75 ± 0.50^b^	1.50 ± 1.29^b^	1.00 ± 0.82^b^
7	200 mg/Kg of chloroform fraction +1 mL p.o of CO	0.75 ± 0.96^b^	1.75 ± 1.26^b^	0.50 ± 0.82^c^	0.50 ± 0.58^c^

Values carrying superscripts different from those of the controls for each hour are significantly different (p < 0.05)

*Effects of the methanol and the chloroform fractions on castor oil-induced diarrhoea in terms of the frequency of defaecation*

Castor oil treatment significantly (p < 0.05) increased the number of stools of the rats in the castor oil-treated control group (group 2) [2.50 ± 0.58, 2.00 ± 0.82 and 1.75 ± 1.26] at the first, second and third hours of post treatment respectively when compared to the values (1.00 ± 0.00, 1.00 ± 0.82 and 0.50 ± 0.58) obtained for rats in group 1 (group treated with vehicle only) as shown in Table 6. The chloroform fraction of the extract at the dose of 200 mg/Kg body weight, like the standard anti-muscarinic drug (hyoscine butylbromide), caused a significant (p < 0.05) decrease in the frequency of defaecation of rats in group 7 (0.75 ± 0.50) at the fourth hour of post treatment when compared to the value (1.50 ± 0.58) obtained for rats in the castor oil-treated control group (group 2). Both fractions of the extract at the tested doses, decreased, in a dose-dependent manner, the frequency of defaecation of rats in group 4, 5, 6 and 7 at the first, second, third and fourth hours of post treatment when compared to those of the rats in the castor oil-treated control group (group 2) as shown in Table 6.

**Table 6:** Effects of the chloroform and the methanol fractions on the frequency of defaecation

**Groups**	**Treatments**	**Number of stools after the**			
		**1** ^st^ ** hour**	**2** ^nd^ ** hour**	**3** ^rd^ ** hour**	**4** ^th^ ** hour**
1	5 mL/Kg of 3% v/v tween 80 (vehicle) only	1.00 ± 0.00a	1.00 ± 0.82a	0.50 ± 0.58a	1.25 ± 0.50a
2	Vehicle + 1 mL per oral (p.o) of castor oil (CO)	2.50 ± 0.58^b^	2.00 ± 0.82^b^	1.75 ± 1.26^b^	1.50 ± 0.58^a^
3	3 mg/Kg of hyoscine + 1 mL p.o of CO	1.25 ± 0.50^b^	1.00 ± 0.82^a^	0.50 ± 0.58^a^	0.25 ± 0.50^a^
4	100 mg/Kg of methanol fraction + 1 mL p.o of CO	2.25 ± 0.96^b^	1.75 ± 0.96^b^	1.50 ± 1.29^b^	1.25 ± 0.96^a^
5	200 mg/Kg of methanol fraction + 1 mL p.o of CO	1.50 ± 1.29^b^	1.50 ± 0.58^b^	0.75 ± 0.96^b^	0.75 ± 0.96^a^
6	100 mg/Kg of chloroform fraction +1 mL p.o of CO	1.75 ± 1.26^b^	1.50 ± 1.00^b^	1.25 ± 0.96^b^	1.00 ± 0.82^a^
7	200 mg/Kg of chloroform fraction +1 mL p.o of CO	1.50 ± 1.29^b^	1.25 ± 0.96^b^	0.75 ± 0.96^b^	0.75 ± 0.50^b^

Values carrying superscripts different from those of the controls for each hour are significantly different (p < 0.05).

*Effects of the methanol and the chloroform fractions on castor oil-induced enteropooling in terms of the weight of intestinal contents*

**Figure 1:**
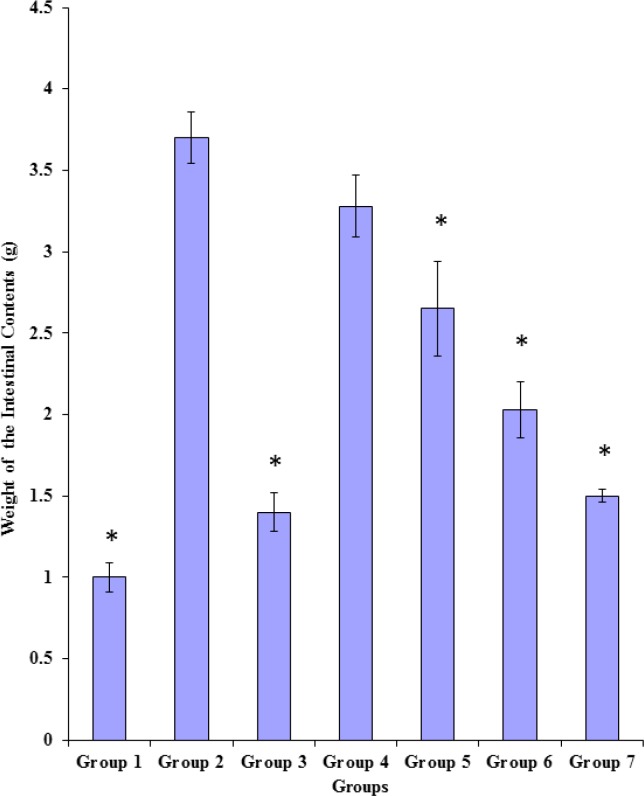
Effects of the methanol and the chloroform fractions on the weight of intestinal contents [Data represented as mean ± SD; *= significantly (p < 0.05) lower compared to group 2].

As shown in Figure 1, castor oil induced significant (p < 0.05) increase in the weight of the intestinal contents of rats in group 2 (3.70 ± 0.32) when compared to the value obtained for rats in group 1 (1.00 ± 0.18) which received only the vehicle. The standard anti-muscarinic drug, hyoscine butylbromide (3 mg/Kg body weight) caused significant (p < 0.05) reduction in the weight of the intestinal contents of rats in group 3 (1.40 ± 0.24) when compared to the value (3.70 ± 0.32) obtained for rats in the castor oil-treated control group (group 2). Both fractions of the extract, at the tested doses, except the methanol fraction (100 mg/Kg body weight), significantly (p < 0.05) and dose-dependently reduced the weight of the intestinal contents of rats in groups 5, 6 and 7 when compared to that of the rats in the castor oil-treated control group (group 2). This effect was comparable to that obtained with the anti-muscarinic drug in rats of group 3. There were significant differences (p < 0.05) between the weight of the intestinal contents of the 200 mg/Kg body weight of the chloroform fraction-treated rats (1.50 ± 0.08) and those of the 100 mg/Kg body weight of the chloroform fraction-treated rats (2.03 ± 0.34), 200 mg/Kg body weight of the methanol fraction-treated rats (2.65 ± 0.58) and 100 mg/Kg body weight of the methanol fraction-treated rats (3.28 ± 0.38) as shown in Figure 1.

Group 1=5 mL/Kg b.w of 3% v/v tween 80 (vehicle)

Group 2=Vehicle + 1 mL of castor oil (CO)

Group 3=3 mg/Kg b.w of hyoscine + 1 mL of CO

Group 4=100 mg/Kg b.w of methanol fraction of *P. americana* +1 mL of CO

Group 5=200 mg/Kg b.w of methanol fraction of *P. americana* +1ml of CO

Group 6=100 mg/Kg b.w of chloroform fraction of P. americana +1 mL of CO

Group 7=200 mg/Kg b.w chloroform fraction of P. americana +1 mL of CO

*Effects of the methanol and the chloroform fractions on castor oil-induced enteropooling in terms of the* *v* *olume of intestinal contents*

**Figure 2:**
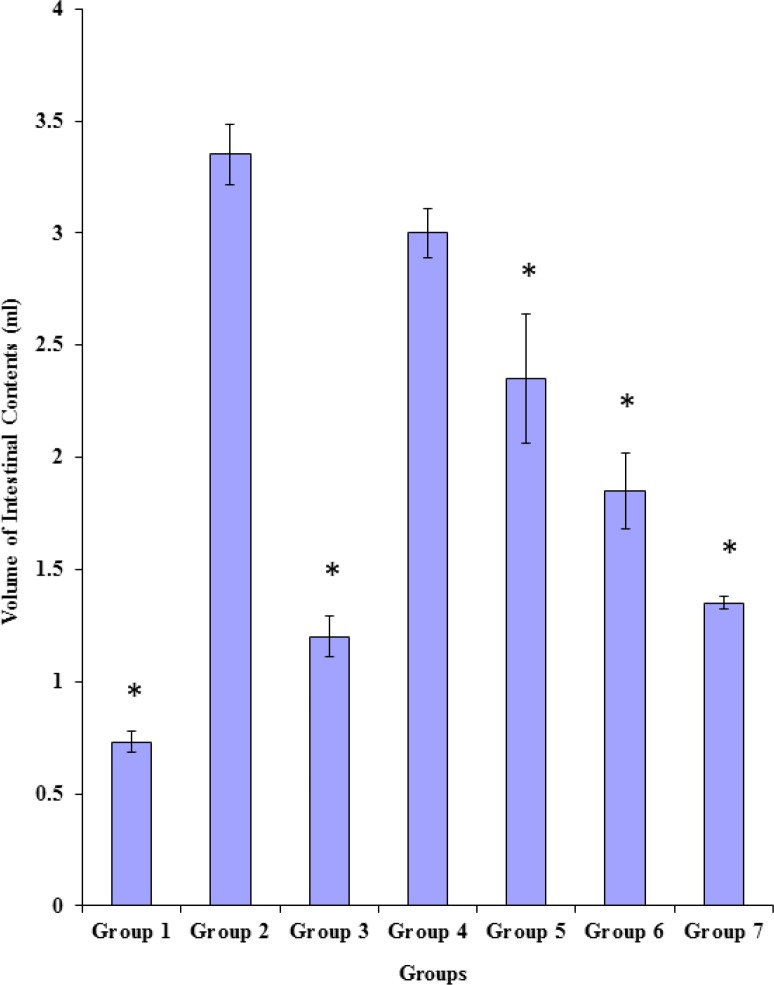
Effects of the methanol and the chloroform fractions on the volume of intestinal contents [Data represented as mean ± SD; *=significantly (p < 0.05) lower compared to group 2].

As shown in Figure 2, castor oil induced significant (p < 0.05) increase in the volume of the intestinal contents of rats in group 2 (3.35 ± 0.26) when compared to the value obtained for rats in group 1 which received only the vehicle (0.73 ± 0.10). The standard anti-diarrhoeal agent, hyoscine butylbromide (3 mg/Kg body weight) caused significant (p < 0.05) reduction in the volume of the intestinal contents of rats in group 3 (1.20 ± 0.18) when compared to the value (3.35 ± 0.26) obtained for rats in the castor oil-treated control group (group 2). Both fractions of the extract, at the tested doses, except the methanol fraction (100 mg/Kg body weight), like the standard anti-diarrhoeal agent (hyoscine butylbromide), significantly (p < 0.05) and dose-relatedly reduced the volume of the intestinal contents of rats in groups 5, 6 and 7 when compared to that of the castor oil-treated control group (group 2). There were significant (p < 0.05) differences between the volume of the intestinal contents of rats in group 7 (1.35 ± 0.06) and those of the rats in group 6 (1.85 ± 0.34), group 5 (2.35 ± 0.58) and group 4 (3.00 ± 0.22) as shown in Figure 2.

Group 1=5 mL/Kg b.w of 3% v/v tween 80 (vehicle)

Group 2=Vehicle + 1 mL of castor oil (CO)

Group 3=3 mg/Kg b.w of hyoscine + 1ml of CO

Group 4=100 mg/Kg b.w of methanol fraction of *P. americana* +1 mL of CO

Group 5=200 mg/Kg b.w of methanol fraction of *P. americana* +1 mL of CO

Group 6=100 mg/Kg b.w of chloroform fraction of *P. americana* +1 mL of CO

Group 7=200 mg/Kg b.w chloroform fraction of *P. americana *+1 mL of CO

## Discussion

Acute toxicity test on the methanol fraction of the chloroform-methanol extract of the leaves of *P. americana* using mice showed an LD_50 _value of less than 5000 mg/Kg body weight. This might be due to the cytotoxic nature of methanol. This indicates that the leaves of *P. americana* might be regarded as being safe to a certain extent with a remote risk of acute toxicity.

Evaluation of the effects of both fractions of the chloroform-methanol extract of the leaves of *P. americana* on diarrhoea experimentally induced with castor oil in rats showed that, they dose-dependently decreased the wetness of faeces and the frequency of defaecation of the treated rats with the effect of the 200 mg/Kg body weight of the chloroform fraction being most pronounced at the fourth hour of post treatment. This indicates that the leaves of *P. americana* contain anti-diarrhoeal agents which exert anti-diarrhoeal effect in a time-dependent manner. However, the chloroform fraction appeared to have decreased the wetness of faeces and the frequency of defaecation more than the methanol fraction (Tables 5 and 6). This might be as a result of the fact that the bioactive constituents responsible for the anti-diarrhoeal effect seem to reside more in the chloroform fraction than in the methanol fraction as shown by the result of the quantitative phytochemical analyses. Also, the finding that castor oil induced diarrhoea in all the castor oil-treated rats is in consonance with the findings of Pierce *et al*. (1971), Zavala *et al*. (1998) and Mynol *et al*. (2008) who observed that the castor oil-induced diarrhoea model in rats allowed for the observation of measurable changes in the consistency and the number of stools. Castor oil induces diarrhoea as a result of the action of ricinoleic acid liberated from castor oil by lipase enzymes. The liberated ricinoleic acid causes irritation and inflammation of the intestinal mucosa leading to the release of prostaglandins which stimulate hyper-motility, alteration in the electrolyte permeability of the intestinal mucosa and increase in the volume of intestinal contents by preventing the reabsorption of sodium, potassium and water (Pierce *et al*., 1971; Galvez *et al*., 1993; Rouf *et al*., 2003). Inhibitors of synthesis of prostaglandins are also known to delay diarrhoea induced by castor oil (Sunil *et al*., 2001). Diarrhoea results from an active intestinal secretion driven predominantly by net secretion of sodium and potassium (Uddin *et al*., 2005). Therefore, the decrease in the wetness of faeces and the frequency of defaecation observed with both fractions of the chloroform-methanol extract of the leaves of *P. americana* in this study are in part, indications of the anti-diarrhoeal effect of the leaves of *P. americana*. This anti-diarrhoeal effect of both fractions of the chloroform-methanol extract of the leaves of *P. americana* might be due to the inhibition of biosynthesis of prostaglandins. 

Both fractions of the chloroform-methanol extract of the leaves of *P. americana* exerted dose-related anti-enteropooling effect in terms of the reductions in both the weight and the volume of the intestinal contents of the treated rats. These observed effects, which are indications of reduced water and electrolyte concentrations in the small intestine, imply that both fractions of the extract probably enhanced the absorption of electrolytes and water from the intestinal lumen, while reducing the rate of their (electrolytes and water) secretion into the small intestine. The anti-enteropooling effect of both fractions of the extract might also be due to the ability of both fractions of the extract to inhibit the castor oil-induced intestinal accumulation of fluid in a manner similar to hyoscine butylbromide (standard anti-diarrhoeal drug). Thus, the anti-enteropooling effect of both fractions of the chloroform-methanol extract of the leaves of *P. americana *in part, could be indicative of an anti-diarrhoeal effect of the leaves of *P. americana*.

In conclusion, the observations above indicate that both fractions of the extract in graded doses reduced diarrhoea by inhibiting wetness of faeces, frequency of defaecation and castor oil-induced enteropooling. These therefore, lend scientific evidence to the use of the leaves of *P. americana* in folk medicine as a remedy for diarrhoea. 
